# Participatory design partnerships for gender and health in low- and middle-income countries: a scoping review

**DOI:** 10.1080/16549716.2026.2627106

**Published:** 2026-04-23

**Authors:** Ulla Walmisley, Gabriella Oliver, Tanya Jacobs, Anam Nyembezi, Olagoke Akintola, Asha S. George

**Affiliations:** a School of Public Health, University of the Western Cape, Cape Town, South Africa; b Institute for Social Development, University of the Western Cape, Cape Town, South Africa

**Keywords:** Gender responsiveness, gender integration, participatory design approaches, partnerships, power relations

## Abstract

Gender inequality remains an intersectional determinant of health insufficiently addressed in programmes and policies. Progress requires systematic integration of gender perspectives but is constrained by the embeddedness of gender inequality across sectors, socioecological levels, and stakeholders. Participatory design approaches hold promise, but the role of participatory design partnerships in advancing gender equality remains underexplored. This scoping review explores what can be learned by examining gender and health programming undertaken within participatory partnerships in low- and middle-income countries (LMICs). Four electronic databases were searched for participatory design and implementation projects addressing an aspect of gender to improve health. Systematic screening resulted in 19 articles published between 2013 and 2023 representing *N* = 17 projects. Data were extracted and synthesised based on intersecting concepts of gender programming, participatory design, and partnership synergy. Across (*N* = 17) projects, (*n* = 6) originated in South Africa, (*n* = 13) focused on sexual and reproductive health or violence prevention, (*n* = 12) used co-design approaches, and (*n* = 11) were gender specific. Participatory approaches complemented gender programming by creating spaces for reflection, adaptation and power-shifting, strengthening participant agency and local buy-in. Most initiatives achieved change at individual or community levels, with limited structural or policy impact. Partnership dynamics were infrequently described. Power dynamics were cross-cutting determinants of project functioning across contextual levels. This review highlights the potential for participatory processes to shift gendered power relations if embedded in inclusive, reflective, and equitably resourced collaboration. Better understanding of partnership processes and synergies between gender integration and participatory design could support future projects advancing gender equality for health.

## PAPER CONTEXT

- **Main findings**: Participatory design approaches can support gender integration in health programming in LMICs but must be situated in inclusive processes and equitable partnerships that consider power dynamics at all stages of projects.
- **Added knowledge**: This review reveals how participatory processes interact with gender and partnership elements in practice, highlighting operational and relational factors that shape outcomes and showing opportunities for future research.
- **Global health impact for policy and action**: Strengthening participatory partnerships through long-term engagement, capacity strengthening, and intentional power analysis can inform more equitable and context-responsive gender policies and programmes, helping to address the root causes of problems affecting population health.

## Background

Strengthened gender equality supports economic growth and improves population health and well-being for men, women, and children [1,2]. There is global recognition of the impacts of inequitable gender norms and power structures [3,4] as these intersect with stratifiers such as socioeconomic status, race, and education [5,6]. High-level commitment to transform the structural underpinnings of gender inequality is evident in its inclusion in the UN 2030 Agenda [7,8], developed in 2015. However, a recent report on progress to address gender inequality for the Sustainable Development Goals found it has been inadequate, citing examples such as failure to meet education targets for girls in Afghanistan, inequitable maternal health outcomes in Madagascar, and persistent gendered poverty in Ghana [9]. These are underpinned by insufficient funding and prioritisation, and subject to resistance and backlash in some contexts [2,10,11]. Policy backlash on gender equality is manifested through legislative measures criminalising sexual rights, and active contestation and removal of gender language in international agreements and global policy documents [12–14]. There is, therefore, a need to continue to advance gender equality while addressing resistance.

Shifting harmful gender norms and power structures is challenging, particularly in health systems. Health systems can be rigid, shaped by people, power structures, and ideologies that are resistant to change [15,16] and which operate in hierarchical environments [17]. The communities they serve, too, are shaped by diverse histories, cultural norms, and power relations [18]. Introducing change, therefore, requires approaches that support stakeholder buy-in across the sectors and societal levels that characterise health systems [19,20].

Participatory design and action can enhance the implementation and sustainability of initiatives by providing opportunities for deeper engagement between stakeholders, creating a sense of joint ownership, and ensuring greater appropriateness of new plans, policies, and programmes [21]. Participatory design and research approaches are based on the philosophy of Paulo Freire, who advocated shifting power to communities, ‘*producing and acting upon their own ideas*’ [22]. These principles of empowerment and participant-generated solutions form the basis of participatory approaches to health system strengthening. They overlap with the notion of shifting power structures, which forms the foundation of gender transformative programming [23]. Collective sense-making inherent in participatory approaches can strengthen buy-in and reduce resistance to power shifting that often accompanies efforts at gender mainstreaming and integration [24,25].

Participatory design collaborations are undertaken within partnerships, whose processes and attributes shape the structure, functioning, and outputs of collaborative endeavours [26,27]. Determinants of partnership functioning include characteristics of partner organisations and how they work together, relationships between partners (the development of trust; the influence of power differentials), and resources (funding, time, and human resources). Meaningful partnerships can support participatory design approaches to challenge and change gendered norms and power structures, so it is important to understand participatory design within the context of underlying partnerships [28].

Given the importance of gender transformative work for health that is owned and sustained over time, there is a need to take stock of how participatory approaches and partnerships have supported gender integration in low- and middle-income countries (LMIC). Additionally, it is necessary to know to what extent these have been transformative, as well as how partnership elements and power relations have shaped these collaborations. This review seeks to answer the question: what can we learn by examining gender and health programming undertaken within participatory partnerships in LMIC settings?

## Methods

**Figure 1.**
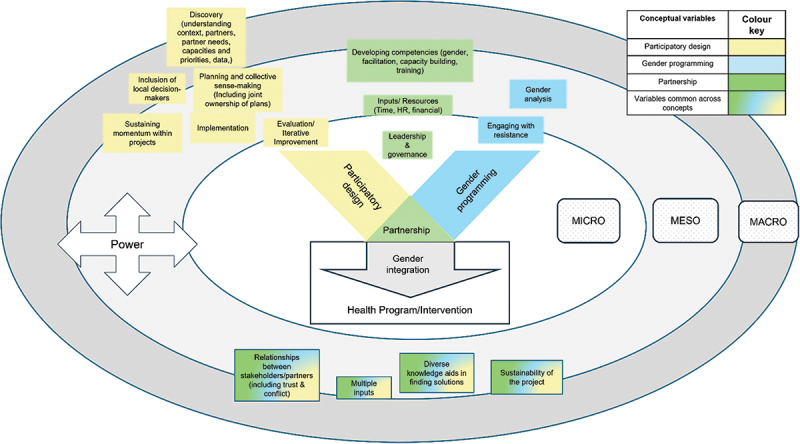
Conceptual framework.

Scoping review methodology was selected to map evidence for this underexplored topic [29]. This review was informed by a framework drawn from three intersecting concepts: gender programming, participatory design and partnership functioning (Figure 1).

Gender programming requires that participants undertake gender analysis [30] and engage with resistance to shifting norms and power relations [25,31]. These processes are supported by iterative participatory design phases of discovery, implementation, evaluation, and improvement [32], through which diverse knowledge is harnessed [33–35] for collective sense-making. This strengthens joint ownership of plans which are more likely to be contextually responsive [36,37]. Both participatory design and gender integration require sustained engagement [38] and trust between participants, to establish an enabling environment for collaboration [19,39]. Gender programming through participatory design is operationalised within partnerships [28], and the eventual shape of gender integration is influenced by determinants of partnership functioning: the quality of relationships between partners, partner and partnership characteristics, and resources employed [26,40,41]. All the above are mediated by the micro, meso, and macro contexts of projects [42,43], and interwoven power relations [30,44]. For example, at the macro level, donor pressures cascade through implementing organisations, leading frontline workers to prioritise targets over addressing the gendered needs of beneficiaries, resulting in box-ticking rather than context-responsive programmes.

This review follows the six-step framework described by Arksey and O’Malley [45] and Levac et al. [29], and adheres to the PRISMA Extension for Scoping Review (PRISMA-ScR) checklist [46].

*Step 1: Specifying the research question*. Specific research questions are:

1. How, where, with whom, and for what purpose have interventions used participatory approaches to integrate gender for health in LMIC?
2. How was gender integrated?
3. How have participatory processes supported gender integration?
4. How did underlying partnerships shape the work of participatory projects?

*Step 2: Identifying relevant studies*: Search terms related to the concepts of participatory design and gender programming (See Supplementary File 1: Search strategy) were developed in consultation with the University of the Western Cape faculty librarian. These were refined and piloted in preliminary searches. This review only included peer-reviewed journal articles in order to explore the state of published literature. A search was conducted for original journal articles published in English between January 2003 and December 2023 across the following databases: CINAHL, PubMed, Scopus, and Web of Science. Search results were uploaded to Covidence for screening.

*Step 3: Study selection*: An abstract screening tool (Supplementary File 2: Abstract Screening Tool) was developed and piloted on an initial set of 20 articles and refined in consultation between G.O., U.W. and A.G. Throughout two-stage screening of 1) titles and abstracts and 2) full texts, G.O. and U.W. reviewed records, with conflicts resolved by A.G. Eligible studies had to be published in English, describe original research projects from LMIC that addressed an aspect of health, include explicit measures to address gender, and include participants as collaborators in design and implementation of the intervention. Studies from high income settings were tagged and excluded. Following consultation, we limited the data set to studies published between January 2013 and 31 December 2023. Extending the timeframe beyond 10 years yielded only one additional eligible article, indicating that the field has grown primarily within the past decade. Exclusions applied to studies that did not address health or gender directly, lacked genuine collaboration beyond consultation, or were formative research without implementation. Reviews, protocols, duplicates, and non-English papers were also excluded (See Table 1).

**Table 1.** Full text inclusion and exclusion criteria.

Inclusion Criteria	Exclusion Criteria
Peer reviewed journal article describing original research	Not original research, including reviews and study protocols
Published in English	Published in a language other than English
Published between January 2013 and 31 December 2023	–
Intervention in an LMIC setting	Non-LMIC setting
Addresses an aspect of health.	Does not address an aspect of health/only addresses a social determinant of health
Uses gender programming as part of the intervention	Does not address gender
Includes participants as collaborators in design and implementation of the intervention	Not participatory design/collaborative (participants are not collaborators in the project)
	Formative research - gathers information but does not apply it or only produces recommendations/guidelines
	Only produces recommendations/guidelines

**Figure 2.**
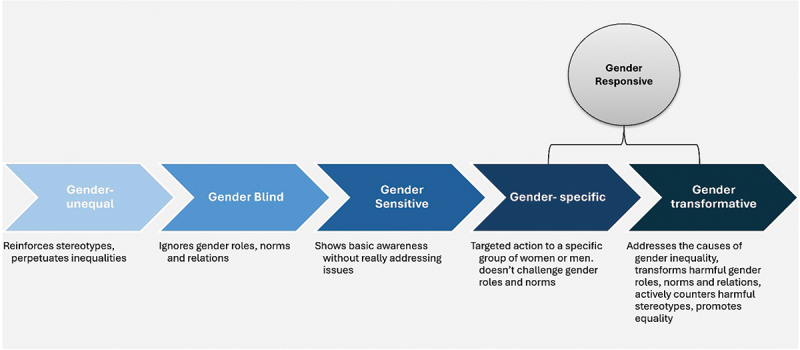
Gender Responsive Assessment Scale. Source: Adapted from World Health Organization [4].

*Step 4: Charting the data*: First, data elements were extracted by GO and UW to describe projects. (Supplementary File 3: Extraction table and samples). Project interventions were categorised for the level at which they operated on the Socio-Ecological Model, namely introducing change at the level of 1) individual knowledge, skills, beliefs, and values, 2) family and peer networks, 3) community, 4) service delivery, 5) enabling environments, and the Gender Responsive Assessment Scale (GRAS) [4] (Figure 2). GRAS describes the extent to which policies and programmes respond to gender inequality. This ranges from gender unequal to gender-specific action (which addresses gender inequality for a specific group but does not tackle its root causes), and to gender transformative action (which includes aims to change norms and power structures more broadly). The latter two categories are regarded as gender responsive. Projects were categorised as gender transformative, gender specific, or unclear (where limited information was provided but gender inequality and the need for shifting gender norms were recognised within the Socio-Ecological Model) (See Table 2).[4]

**Table 2.** Overview of projects’ country of origin, gender dimensions, aims, approach, stakeholders & duration.

**Projects identifier: First author of papers, year**	**Country & health focus**	**Gender Dimension**	**Aim of projects**	**Brief description of project processes**	**Participatory Approach**	**Participants/Stakeholders**	**Study duration**
**1. CO-DESIGNED PROJECTS**							
**Artz, 2017**	**South Africa** Gender-based violence (GBV)	Gender specific	To develop and pilot the Z-card: a tool to assist survivors in managing their health and criminal justice outcomes after a sexual offence. This was to be done in partnership with implementing organisations and feedback from users/beneficiaries.	Researchers and non-governmental organisation (NGO) partners agreed on the project’s value and contributed to its design. Two township-based organisations were to distribute numbered Z-Cards to survivors during first counselling sessions, track their reuse in follow-up visits, and enrol 350 survivors for phone interviews on the cards’ usefulness in navigating health and justice systems. Researchers committed to producing and supplying the Z-Cards and generating evidence for broader funding and distribution. However, limited partner capacity and difficulties in contacting survivors created barriers.	Feminist participatory research	Researchers and service provider organisations	Unclear
**Bankar, 2018 Collumbien, 2019 (Parivartan Project)**	**India** Physical functioning (participation in exercise)	Gender transformative (Worked on multiple levels and with stakeholders)	The Parivartan Project aimed to address harmful norms related to restriction on girls’ liberty to move freely in public spaces and contested discriminatory norms while implementing a sports-based programme for adolescent girls in a Mumbai slum.	Young women from the community took part as co-designers of the programme during reflective workshops which shaped strategies for negotiating movement and engagement in public spaces. Continued feedback helped the intervention to adapt and led to a more contextually appropriate curriculum, and programme delivery. Design engaged community leaders and families to address and challenge resistance, and positioned mentors as programme leaders.	Participatory Action Research (PAR). No specific method	Families, girls, community NGO	15 months
**Beeman, 2023**	**Uganda** Menstrual hygiene	Gender specific	To co-design the Cocoon Mini, a safe, physical structure for managing menstruation, while engaging men in dialogue around sexual and reproductive health and reducing stigma.	This project aimed to co-create safe, dignified, and context-specific menstrual health spaces in displacement settings by engaging adolescent girls and women, as well as men and the broader community, in a 5-phase design process . While the primary goal was to improve menstrual hygiene infrastructure, the process also challenged social taboos and encouraged dialogue about menstruation.	Human-centred design	Research and design organisation, a non-profit organisation (NPO), a global data collection firm, and community members from a refugee camp: people who menstruate, male community members, other community stakeholders	1 year
**Chowdhary, 2018**	**Syria** Adolescent sexual and reproductive health (ASRH)	Gender specific	To collaboratively create and implement the Adolescent Mothers Against all Odds (AMAL) Initiative for pregnant girls and first-time mothers aged 10 to 18 years. Designed to improve the lives of young girls through responsive health systems and enabling environments, AMAL included three components: a Young Mothers Club for first-time mothers and pregnant girls, participatory dialogues with health providers, and reflective dialogues with girls’ marital family and community members.	The programme centred on married adolescent girls as active participants in shaping reproductive health services, by creating safe spaces for girls to share experiences, analyse power dynamics in their lives, and engage with service providers and community stakeholders. This process empowered the adolescents to voice their reproductive health needs, challenge age- and gender-based discrimination, and influence decision-making within their families and health systems.	PAR (no specific method)	Girls, health providers, girls’ marital family and community members	1 year
**Doan, 2022**	**Vietnam** HIV/AIDS Sexual and reproductive health (SRH) of a minority group	Gender specific	To improve the quality of PrEP services reaching transgender women as part of integrative primary health care in Vietnam, through collaboration with the transgender community, health workers and other partners.	This intervention integrated PrEP services into primary healthcare specifically tailored to the needs of transgender women. It emphasized respectful, affirming care by training healthcare providers in transgender-competent service delivery, addressing structural stigma, and offering wraparound services like hormone therapy and mental health support. The model was developed through community-based partnerships and emphasized person-centred care that recognized transgender women’s specific vulnerabilities and identities, and led to input on government guidelines for trans care.	Participatory continuous quality improvement (CQI) & Plan-Do-Study-Act cycle	Vietnam ministry of health, USAID, CQI teams around each clinic consisting of health workers, managers, community PrEP users, local government HIV focal points and project staff	4 years
**James, 2021**	**Malaysia, Lebanon** GBV prevention	Gender specific	To co-design and test two community-based GBV interventions in humanitarian settings.	The programme engaged both women and men in gender-segregated and mixed sessions, using facilitated discussions to unpack harmful norms, explore power dynamics, and build empathy and communication skills. The community-based approach was tailored to each cultural context and aimed to shift the underlying social norms that sustain gender inequality.	Co-design	Researchers, partner organisations, Community Advisory Committees (including community leaders, religious leaders, civil society leaders, and teachers)	4 months
**Kutwayo, 2018**	**South Africa** ASRH & GBV	Little information: recognises gender inequality and norms shifting needs, based on Socio-Ecological Model	To plan, implement and evaluate a curriculum with stakeholders including adolescents, parents, teachers, health workers, and government officials to empower adolescent girls and shift gender norms by improving their educational, health, social, and economic opportunities.	The approach emphasized co-creation, ethical partnership, and power-sharing between researchers and adolescent girls, particularly those most marginalized. Girls were involved as peer researchers, advisors, and advocates, shaping research agendas and outreach strategies. By embedding principles of respect, agency, and inclusion, the intervention moved beyond consultation to true collaboration, challenging adult-centric power.	PAR (no specific method)	Government departments, school governing bodies, civil society, adolescent girls	2 years
**Malta, 2023**	**Brazil** GBV	Gender specific	To develop a mobile health intervention (the Dandarah app) to address violence and discrimination against sexual and gender minorities, in collaboration with this population.	The project used in-depth interviews and focus group discussions - the initial phase gathered information from community leaders, key informants and service providers for sexual and gender minority (SGM) persons experiencing violence and discrimination. Thereafter the app was developed and pilot tested with SGM community members. A community advisory board of SGM members collaborated with the research team to develop culturally sensitive and adequate recruitment strategies, adapt study protocols and tools, and contribute to interpreting and disseminating research findings.	Community-based participatory action research (CBPAR)	Brazilian SGM community, healthcare professionals, researchers, and government representative	1 year
**Mauka, 2021**	**Tanzania** HIV/AIDS SRH of a minority group	Gender specific	To support PrEP adherence among female sex workers (FSW) and men who have sex with men (MSM) in Tanzania, through the collaborative development of a mobile health (mHealth) app.	This intervention recognized gendered vulnerabilities and tailored services for women and men. The design process was participatory, engaging users from these communities to ensure the app addressed their specific needs, including privacy concerns, stigma reduction, and access to resources. The app provided health information, facilitated testing services, and offered support for safer sexual practices.	Participatory design based on the information system research framework (ISR)	MSM and FSW, researchers, software developer	Unclear
**Moran, 2022**	**Pakistan** COVID-19 prevention	Gender specific	To co-design and implement a culturally appropriate COVID-19 risk communication and community engagement strategy with a resource-poor rural community in Northwest Pakistan.	The process involved community members, including women and men, in identifying local knowledge, beliefs, and practices related to COVID-19. The strategy focused on addressing gendered barriers to information access and decision-making, ensuring that women (particularly those in rural settings), could actively participate in shaping the public health response. It integrated women’s voices in health communication, aiming to improve their understanding of health guidelines and their ability to share this knowledge within their households.	PAR	Government representatives, schools, NGO, community members	8 months
**Stern, 2021**	**Peru, Rwanda** GBV	Gender transformative	The GAP Project used a Participatory Community-led Intervention Development (PCID)approach to work collaboratively with local promotores (community health workers) to identify risk factors for GBV and develop a set of targeted prevention activities for their communities.	In Peru, the Gender Violence in the Amazon of Peru (GAP) Project engaged community health workers (promotores) through a participatory, community-led process. They co-designed and implemented local GBV prevention strategies across remote communities in a rural region. Activities included workshops, role plays, community meetings, and creative media, developed using PAR principles.	PCID	Community activists, NGOs, researchers	1 year
**Varjavandi, 2017**	**South Africa** ASRH	Categorisation is unclear, recognises gender inequality and norms shifting needs, based on Socio-Ecological Model	To investigate the use of visual methods in youth-led PAR and their potential in exploring resilience-enabling factors in the context of gender inequality	#Blessers Must Fall intervention was a youth-led project conducted at a secondary school. Youth determined the issues most relevant to their lives and collaboratively developed responses that included public performances and photo story posters. The intervention unfolded in six iterative phases - pre-reflection, topic identification, data gathering, planning, implementation, and evaluation - with an emphasis on co-constructing knowledge. It used creative and visual methods to explore intersecting challenges of teenage pregnancy, transactional sex (the ‘blesser’ phenomenon), and GBV.	PAR (no specific method)	Researcher, adolescents	Unclear
**2. CO-CREATED PROJECTS**							
**Ahlberg, 2015**	**Kenya** HIV/AIDS	Flags gender power dynamic but can’t categorise on the continuum. Researchers note the need to understand gender power dynamics, due to resistance from men.	Building on a project which focussed on HIV/AIDS prevention among school youth in Kajiado in Kenya during 2003–2006, this project used community-led planning to improve water supply - as a way to engage men and community members in dialogues about SRH and social issues (nutrition, child resilience, development of community empowerment).	This project used the action of planting trees (and associated plans to access a reliable and safe water supply), and to create space for open dialogue about gender, sexuality, fertility, and reproductive rights. It engaged the community in reflective processes to question traditional norms and power relations around fertility, childbearing, and gender roles.	PAR (no specific method)	School youth, parents, researchers, international funders, NGO, engineer	7 years
**Forbes-Genade, 2019**	**South Africa** Adolescent psychological health (resilience)	Gender specific	The GIRRL Programme used a PAR approach to help reduce the vulnerability of adolescent girls in a resource-poor setting by co-creating a programme of strategic activities for a more inclusive disaster risk reduction environment.	This project supported adolescent girls to identify and act on issues affecting their safety, health, and rights within their communities. Through facilitated workshops and collective inquiry, girls explored the root causes of gender-based violence, poverty, and marginalization, and designed community actions to challenge these issues. The intervention placed girls in leadership roles, promoted critical consciousness, and fostered solidarity, resilience, and advocacy skills.	PAR (no specific method)	Stakeholders included representatives from Community Health, Public Safety, Police, Fire, Education, Environmental Health, Disaster Management, North-West University, Love Life (HIV/AIDS NGO), local councillors, and community leaders. Key participants were youth centre managers, Love Life coordinators, social workers, firefighters, police, disaster centre officials, and school principals and life skills teachers.	17 weeks
**Pepper, 2023**	**South Africa** HIV/AIDS	Gender specific, empowerment of women	To address barriers to HIV treatment adherence by postpartum women living with HIV, using an inclusive community-led approach	Ten women co-researched and documented their lived experiences of anticipated HIV stigma, extreme poverty, and gender-based constraints. Their work culminated in the Lelethu Programme, a locally grounded, community-led intervention developed in partnership with a local NGO (Ubunye Foundation). The programme addressed stigma, economic disempowerment, and psychosocial distress through multiple components: personal “Life Plans,” peer support groups, community advocacy, and economic mentoring.	CBPAR	Health care workers, NGO, women	4 years
**Kerr 2016 Patel, 2015 (Recipe Days project)**	**Malawi** Child nutrition	Gender transformative	This integrated agricultural and nutrition education project sought to improve nutrition in a resource-poor agrarian setting by supporting gender equality. The project aimed to transform inequality by facilitating men’s engagement in productive and reproductive tasks, promoting development of new masculinities.	The programme promoted household-level food production and dietary diversity, while incorporating sessions that encouraged shared decision-making between men and women regarding food allocation, labour, and income. While initially designed to improve nutrition outcomes, the intervention recognized gendered barriers - such as unequal control over resources and time burdens - and introduced strategies to promote greater gender equity within households.	CBPAR	Researchers, community members	7 years
**Taliep, 2023**	**South Africa** Inter-personal violence prevention (IPV)	Categorisation is unclear, recognises gender inequality and norms shifting needs-based Socio-Ecological Model	The aim of this paper is to describe and reflect on community asset mapping as a PAR modality for developing an IPV programme that focused on the promotion of positive forms of masculinity to foster safety and peace.	This project engaged local residents, service providers, and faith-based groups to identify tangible and intangible community assets, prioritize safety-related needs, and co-design solutions. The intervention focused on promoting positive forms of masculinity as a means of fostering peace and safety, challenging harmful gender norms, and addressing structural drivers of violence - with a mentorship programme as the central intervention.	CBPAR	NGO, community members, government representatives, religious organisations, civil society organisations	Unclear

Participatory approaches were categorised according to the Vargas [47] definition (Box 1). *Box 1: Definitions of participatory design*

Box 1: Definitions of participatory design

- *Co-creation: Collaboration from the stage of problem identification and solution generation to implementation and evaluation.*
- *Co-design: Active collaboration between stakeholders in designing and implementing solutions to a pre-specified problem.*
- *Co-production: Implementing a previously determined solution to a previously recognised problem with participant collaboration limited to adaptation for contextual appropriateness.*

**Source**: Vargas et al. [47]

Next, data were extracted for each project using an Excel extraction table based on the conceptual framework and piloted on 10 articles. Variables were grouped under each concept within the conceptual framework, including a category where variables were common to multiple concepts. Power was a cross-cutting element.

*Step 5: Collating, summarising, and reporting the results*: A numeric analysis of descriptive aspects of studies was carried out. Thereafter, text excerpts were analysed using deductive and inductive thematic analysis to identify patterns. They were then grouped so that themes aligned with concepts: gender dimensions of projects; participatory design approaches, partnership dimensions of projects, with power emerging as a new and separate theme.

*Step 6: Consultation*: Team members of a co-design project (the Learning Partnership for Gender Transformation) were consulted to validate data extraction categories and ensure we captured salient elements of participatory design for health, based on their experience of working in such a partnership.

## Findings

### Description of included records

**Figure 3.**
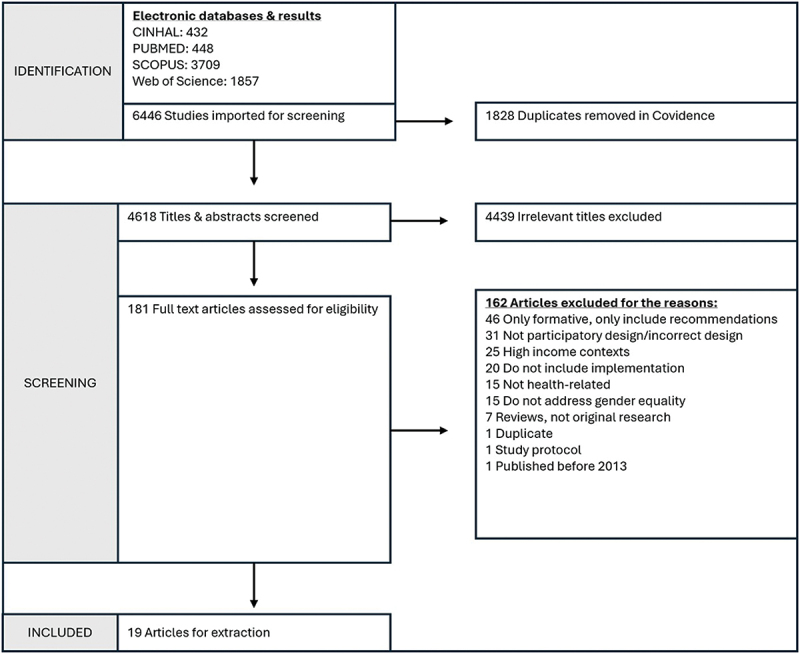
PRISMA flowchart.

Figure 3 provides a PRISMA flowchart for the selection of studies. Table 2 gives an overview of included projects.

A systematic search was carried out, yielding 6446 articles from 4 electronic databases from which 1828 duplicates were automatically removed by Covidence. A total of 4618 titles and abstracts were screened, with 4439 studies found to be irrelevant which produced 181 full text studies. Systematic screening excluded 162 studies leaving a final set of 19 articles, representing 17 participatory design projects. Two projects are represented by two articles each: Kerr et al. [48] and Patel et al. [49] describe the ‘Recipe Days’ initiative in Malawi. Bankar et al. [50] and Collumbien et al. [51] describe the Parivartan project in India.

### Characteristics of projects

**Figure 4.**
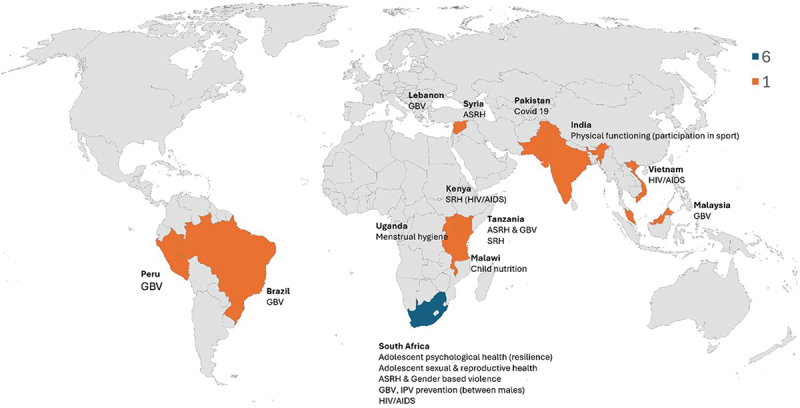
Global distribution and health focus of projects.

Table 3 shows the characteristics of included projects, including context, health focus, design approach, and GRAS categorisation. Figure 4 shows the global distribution of included projects and the health issues they addressed.

**Table 3.** Characteristics of included projects.

NUMBER OF PROJECTS		(*N* = 17)
World Bank Country Classification*	Country	(n)
Upper-middle-income	South Africa	6
	Brazil	1
	Malaysia	1
	Peru	1
Lower-middle-income	India	1
	Kenya	1
	Lebanon	1
	Pakistan	1
	Vietnam	1
Low-income	Tanzania	1
	Malawi	1
	Syria	1
	Uganda	1
**Context/settings**		
Urban area(s)		8
Rural area(s)		5
Humanitarian setting		3
Multiple areas, not described in detail		1
**Health Focus**		
**Sexual and reproductive health & Violence**		(*n* = 13)
Gender-based violence		4
Sexual and reproductive health (any age) *Includes HIV/AIDS		4
Adolescent sexual and reproductive health & gender-based violence		2
Adolescent sexual and reproductive health *Including HIV/AIDS		1
Inter-personal violence prevention (between males)		1
Menstrual hygiene		1
**Other health issues**		(*n* = 4)
Adolescent psychological health (resilience)		1
Child nutrition		1
COVID-19		1
Participation in sport & physical functioning		1
**Participatory design approach**		
Co-design		12
Co-creation		5
**GRAS categorisation of projects**		
Gender specific		11
Categorisation is not clear		3
Gender transformative		3
**Project stakeholders/actors**		
**Category**	**Subcategory**	**Instances of inclusion in projects**
**Civil society, NGO, NPO**	Religious	5
	NGO/NPO/Civil society	15
**Public**	Whole community	7
	Family/household groups	5
	Advisory groups	4
	CHW/Champions	3
**Researchers**		18
**Programme beneficiaries**	Adult beneficiaries	6
	Youth beneficiaries	8
**Service provider**	Schools	4
	Health	6
	Law enforcement, social services, support	2
**Government**	Government departments (Education, health, social development), councillor	5
**Specialists consultants**	Software developer, engineer	2

*Note. Country income classifications are based on the World Bank’s World Development Indicators [52].

*Setting -* Most projects were situated in urban areas (*n* = 8) [50,51,53–59], rural areas (*n* = 5) [48,49,60–62] and humanitarian settings (*n* = 3) [39,63,64].

Global distribution – Most were from South Africa (*n* = 6) [53,55,58,61,65], and the remainder had one study each from Tanzania [57], Syria [63], Brazil [56], Kenya [60], Malaysia and Lebanon [64], Pakistan [62], Uganda [39], India [50,51], Malawi [48,49], Vietnam [54] and Peru [66].

*Health focus -* Most projects addressed sexual and reproductive health and/or violence (*n* = 13). Only four studies addressed other health issues: one each for adolescent psychological health and resilience [61], childhood nutrition [48,49], COVID-19 [62], and participation in sport [50,51].

*Participatory design approach* - Five projects were co-created with community participants, who were engaged in identifying the health problem that was addressed. Plans were then designed and implemented [48,49,58,60,61,65]. The rest (*n* = 12) were co-designed, meaning that the problem was identified first by researchers or other organisations, following which communities or target groups participated in designing and implementing interventions.

*GRAS categorisation for gender responsiveness* – (*n* = 11) projects were gender-specific in that they responded to specific gender needs and lived realities of beneficiaries, but did not address underlying causes of marginalisation [39,53–57,61–65]. *n* = 3 projects were gender transformative and addressed root causes of inequality [48–51,66]. *n* = 3 projects could not be clearly categorised based on the information provided: while they engaged with gendered issues across different levels of the socioecological model, the extent to which they were gender-specific or transformative was not sufficiently defined [58–60].

**Figure 5.**
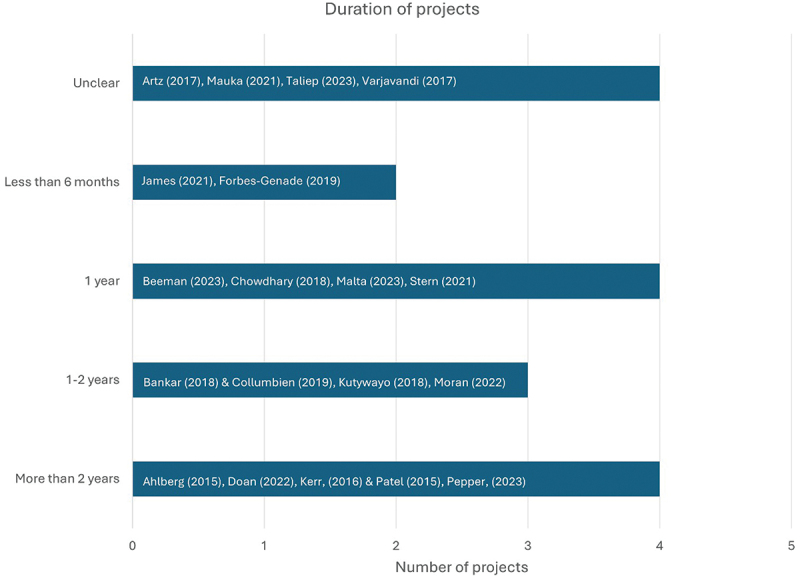
Duration of projects showing author names.

*Project duration –* The duration of (*n* = 4) projects was unclear based on information provided. (*n* = 13) projects reported durations ranging from 4 months to 7 years (Figure 5). Of projects that were longer than 2 years, two operated for 4 years, and two for 7 years [48,49,54,60]. These longer project durations were enabled by long-term funding commitments and multisectoral collaborators (government organisations, universities, international and local NGOs) with established relationships with communities.

**Figure 6.**
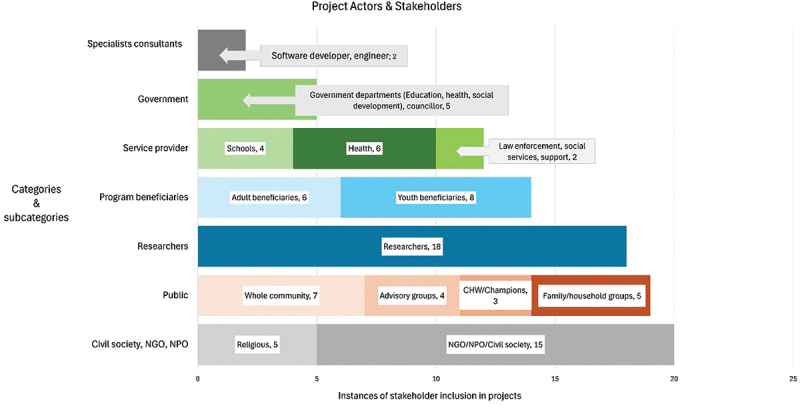
Categories and sub-categories of project actors and stakeholders.

*Project actors and stakeholders –* Projects had varying participant compositions (Figure 6). These were predominantly programme beneficiaries, civil society and NGOs, researchers, general public participation (which included whole communities), advisory groups, community health champions, government representatives (representatives of national or local departments such as health, education, social development, or ward councillors), service providers – and in two cases – consultants who provided technical input or services.

### Narrative description of project elements

This section describes elements of gender programming, participatory design, and partnership functioning based on data extracted from 19 articles representing 17 projects. A brief overview of each project – including its country of origin, gender focus, aims, participatory approach, key stakeholders, and duration – is presented in Table 2, which serves as an introduction to the projects discussed here.

#### Gender programming

This section examines how programming to address gender inequality was understood through gender analysis, socioecological levels of change, and experiences of resistance.

##### Gender analysis

Most authors do not explicitly describe their problem analysis as a ‘gender analysis’ and none used a specific gender analysis framework. Projects considered gender in varying ways, examining gender inequality in programme content, process and/or outcomes. Projects anticipated how gendered power dynamics could shape content (influencing participation in programmes) [50,51]. Additionally, projects anticipated the extent of the use of services and facilities [55,63] in project processes (by considering how gendered power relations shaped research) [57,62,65] and in outcomes (aiming to transform power relations) [39,58,63,65]. Only two projects recognised gender as being more than binary: a menstrual health project in Uganda (which used non-binary language) [39] and a project supporting trans-competent services [54].

##### Level of change

Comparing interventions to the socioecological model shows that two projects aimed to improve service delivery by 1) developing an mHealth app to strengthen ARV adherence [57] and 2) training healthcare workers to deliver adolescent-friendly services [63]. One project reported action that could result in policy change by developing national HIV and transgender healthcare guidelines with the Vietnamese Ministry of Health to support scale-up of services [54]. The others aimed to shift norms in individuals, families and communities.

##### Resistance

Gender programmes described resistance to shifts in power or challenges to traditional norms (*n* = 7) and used various strategies to deal with it. In Peru, male health promoters described negative perceptions by communities for transgressing gender norms in their role as activists countering GBV [66]. Strategies to counter this included the use of empathetic and respectful communication, securing partnerships beyond the community (including with government actors) and forming alliances with local leadership structures.

In India, planners in a programme that used sport as a ‘hook’ to change norms about female participation in physical activity and engagement in public life faced opposition when parents realised girls would need to walk through communities for 45 minutes to reach the venue. To reduce resistance, they partnered with an NGO that had a long-established history in the community, a relationship of trust with community members, and insight into contextual realities. They identified ‘positive deviant’ families who were likely to be receptive to norm shifting, in order to avoid potential resistance by not having to seek male permission for girls to act as change agents. Programme mentors served as examples of culturally acceptable behaviour change who could ‘translate new ideas to parents’ [50,51].

The ‘Recipe Days’ intervention in Malawi faced resistance to men participating in childcare and cooking. This was reduced when men and women participated in workshops characterised by cultural engagement (singing and dancing) and collective activity (preparing food together) over a sustained period [48,49].

Other projects encountering resistance sought to include influential groups (particularly male religious and community leaders) and used intergenerational involvement (parents, mothers-in-law, siblings, husbands) [63], and engagement of men and boys as well as women to increase buy-in [39].

Efforts to change the status quo sometimes entrenched it. In a humanitarian setting, patriarchal norms were reinforced [64], while in Peru a project addressing gender-based violence (GBV) found that women did not have time available to work as health promoters. It was men, therefore, who became GBV experts in the community - limiting input from women’s perspectives and further entrenching men taking leadership roles [66]. The above examples demonstrate the importance of establishing relationships and a deep understanding of contextual dynamics in programme design.

#### Participatory processes

Participatory processes were supported by 1) pooling diverse knowledge, 2) iterative, flexible engagement, and 3) training, support, and capacity strengthening. Participation created space for jointly owned solutions and collective engagement

##### Pooling diverse knowledge

Gender programming benefitted from participatory processes that were inclusive of knowledge drawn from diverse sources, including community participants, stakeholders, and specialist expertise [57,60]. For example, a project in South Africa co-created a violence reduction plan with input from an NGO, community members, faith-based organisations, local government representatives, and service providers. This led to the development and implementation of a mentoring programme to promote positive masculinity, showing how such multisectoral action can enable communities to address gender inequality that underpins contextually relevant health issues [58].

Projects encountered challenges to the inclusivity of some participant groups: Cultural norms hindered male involvement in discussions of sexual and reproductive health [39,60,66]. Ethical issues challenged engagement with vulnerable groups such as younger adolescents and survivors of sexual assault [53].

##### Iterative, flexible engagement

Knowledge gained during multistakeholder participation processes fed into sense-making and planning, usually characterised by iterative collaboration. Projects (*n* = 15) describe iterative change during design and implementation, which supported project aims by increasing contextual responsiveness or surfacing unanticipated issues. (Flexible adaptation of plans is described in Supplementary File 4.) Examples of flexibility include a project which addressed menstrual hygiene in refugee camps. The project used human-centred design with five phases: background research; design research; rough prototyping; live prototyping; and piloting. The aim was to develop and implement culturally and contextually appropriate plans [39] In other projects, iterative changes occurred organically, illustrated by the Recipe Days intervention in Malawi. This initiative developed during an agricultural programme that struggled to achieve its goal of reducing childhood malnutrition. Over time, the community identified a specific issue: while women had access to information on child nutrition, they did not have the power to put that knowledge into effect without buy-in from men [48,49]. As the project progressed, flexibility was important in shifting the focus of the project onto dynamics around gender and power.

Adaptation and improvement stemming from sustained engagement is described by the Parivartan project in India. This initiative to shift norms around female physical activity and participation in public life found that ‘ongoing interactions with stakeholders during the implementation stage further guided adaptations to overcome local barriers’ [51], thus supporting implementation.

##### Training, support, and capacity strengthening

Participants assumed new roles as co-researchers [65], advisors [54,63], mentors [50,51], facilitators [66], leaders, and decision-makers [65]. Capacity strengthening was seen as a promising way to strengthen communities’ independence – noted by authors addressing menstrual hygiene and sexual and reproductive health in a humanitarian context in Uganda. They found that capacity strengthening could ‘potentially decrease reliance on NGOs and aid partners, while promoting skill-building and independence in humanitarian contexts’ [39]. Table 4 gives details of training within projects.

**Table 4.** Training within projects.

Bankar et al. 2018 & Collumbien et al. 2017 – India	Female mentors trained in mentorship, facilitation, delivering a norms shifting curriculum, skills to negotiate greater mobility in communities, sports coaching for *kabbadi*.
Beeman et al. 2023 – Uganda	Twenty community supervisors trained to oversee menstrual health structures, including safety and feedback.
Chowdhary et al. 2018 – Syria	Adolescent girls trained as leaders; health providers trained in adolescent-friendly SRH and long-acting contraception.
Doan et al. 2022 – Vietnam	Clinic staff and community-based organisations trained in gender-affirming, transgender-competent HIV care.
Forbes-Genade 2019 – South Africa	Girls trained in participatory action research, facilitation, leadership, and resilience-building.
James et al. 2021 – Syria	Facilitators trained in participatory and social norms methods; participants co-created poster campaigns.
Kutywayo 2018 – South Africa	Facilitators trained in participatory practice, safeguarding, and reflexive learning.
Malta 2023 – Brazil	Facilitators/community workers trained in rights-based, anti-discrimination support for LGBTQ+ persons.
Mauka 2021 – Tanzania	Peer educators and health workers trained in digital literacy and app use for HIV prevention.
Pepper 2023 – South Africa	Postpartum women trained as co-researchers via Photovoice; capacity building in analysis and programme co-design.
Stern 2022 – Peru	Activists trained and mentored in gender, power, facilitation.

Training and support were required by facilitators and mentors, who played an important role in bridging the divide between partner organisations and communities for programme delivery. Fulfilling this role required capacity strengthening through structured training and support [50,51,64,66]. A Peruvian study describing the implications of its findings notes ‘the critical role that (programme facilitators) play in the success of community mobilisation efforts, and the importance of careful and inclusive recruitment, offering sufficient training that responds to capacity gaps (i.e. facilitation skills, psychosocial counselling) and regular support’ [66].

##### Jointly owned solutions

Inclusive processes led to collective ownership of creative and contextually appropriate interventions in (*n* = 15) projects (Supplementary File 5 describes joint ownership of plans). As expressed by a project participant in a Brazilian intervention that developed an app to safeguard trans people, ‘… this participation of the ENTIRE LGBT community is just … Amazing. I mean, this makes all the difference. That’s not a study that some white, rich researcher planned and decided to do. Do you know what I mean? That’s something built for and by our community. And OMG, I’m so proud to be here! We’re making history!’ [56, p.4]. This reflects a sense of shared ownership and support for the intervention. In Vietnam, multisectoral collaboration which included the trans community successfully increased PrEP uptake [54], showing how partnership with a marginalised group reduced stigma and supported service delivery.

##### Joint engagement

Most projects engaged men as well as women. (Supplementary File 6 describes gendered engagement). Only one project solely engaged women: Pepper (2023) which focused on postpartum women living with HIV/AIDS [65].

Group engagement enabled participants to adopt roles as planners, leaders, and mentors. In the study by Pepper (2023) mentioned above, women who ordinarily faced stigma and marginalisation co-created plans and actions for personal development and capacity strengthening [65]. They became advocates in their community, furthered their education, accessed economic opportunities, and shared their work in a public forum at the French Embassy in Cape Town [65]. Prior to this, they had been a marginalised group in their community. During collective engagement, joint engagement created opportunities for participants to “try on” new roles. As expressed in a project from Malawi: ‘Recipe Days provide a space where men can feel safe and encouraged in new performances of “counter-hegemonic masculinity”, and where women can subvert gender roles and “undo gender” with a freedom hard to find in other spaces. The creation of these spaces makes it easier for gender transformation to occur’[49]. In Pakistan, women engaged in a public forum as decision-makers, which ran counter to community expectations regarding their roles. The above examples indicate the value of group engagement as a transformative space.

#### Partnership

The participatory nature of projects meant that they were shaped by elements of partnership synergy: relationships between partners (how connections and trust enabled collaboration and reduced conflict between partners), partnership characteristics (characteristics of partners and how they worked together); power dynamics (leadership and governance); and resources required for the partnership to function (financial, time and human resources).

##### Relationships between partners

Relationships between project participants, stakeholders, and researchers were described as a factor that supported collaborative work. Researchers were often strategic, partnering with established organisations that had long-standing relationships of trust with communities [48–51,55,56,58,60,66]. Projects recognised the importance of social capital [66] and were intentional about building relationships through community meetings [39], cohesion-building activities [64] and fostering connections with local leadership [66] and stakeholders [58]. This allowed for participant and community buy-in and dialogue but reduced potential negative consequences that could arise from trying to shift power, as described in this norm-shifting intervention in India: ‘With a relationship of trust built over 40 years of community development, Apnalaya could gauge the extent of risk that was acceptable’ [51].

Positive aspects of relationships are widely described, but conflict is rarely referred to except for ‘audacious differences in opinion’ – when strong disagreements surfaced within the academic–community research team (related to project roles and priorities, and contrasting perspectives on priorities and assets). This required sensitive handling [58] to avoid tension between researchers and NGOs who managed funding on their behalf [60]. In a project addressing water supply as part of a programme to open dialogue around SRH, the community was clear about what they needed. Funding was given by Global North donors to an intermediate organisation, who acted as a gatekeeper and had to be convinced of the merit of project components before funds could be disbursed. They ultimately funded alternative plans which faltered [60].

##### Partnership characteristics

Although partner organisations and communities are likely to have quite different characteristics, the way in which these complemented each other or created challenges is not much described. Exceptions are projects 1) which reported how pressurised work environments undermined an undertaking in which all partners were enthusiastic at the outset, ‘… although we had a long working history with the two NGO collaborators and they were eagerly involved in the research project from conception, during the process, the organisations made strategic choices based on the immediate demands of their service provision role at the expense of the research project’ [53], and 2) where the democratic nature of the community-led project was at odds with the hierarchic nature of funding organisations [60].

Governance and leadership within projects are not much described apart from a project working to build resilience with teen girls in South Africa, which reported that taking on a role ‘managing’ the project was inherently problematic: ‘Financially and administratively, the “academic actor” becomes responsible for reporting on the programme (which) could undermine the autonomy of the participants.’ [61], because it gave researchers greater decision-making power.

##### Resources

Descriptions of resources are largely limited to financial resources, with little mention of time or human resources.

###### Financial resources

Funding is discussed as an opportunity for power shifting, an enabler of project implementation, as well as a constraint and a source of tension.

###### An opportunity for power shifting

– Moran (2022) describes a COVID-19 risk-mitigation project in Pakistan. Community members were allocated a fixed budget of £40,000 to implement co-designed strategies according to self-identified priorities. This enabled them to ‘go beyond discursive reflection on issues facing the community and involve themselves in democratically deciding how resources could be mobilized’ [62].

###### An enabler of project implementation

– Stern (2021) emphasises the need for adequate funding to meet the material needs of activists, enabling them to do their work without financial burden or hindrance.

###### A constraint to collaboration

–Ahlberg (2015) found that global funding arrangements resulted in an intermediary organisation choosing to fund a solution to water scarcity that had not been developed by the community affected. It ultimately failed. Funding intersected with cultural, gender, and material power to constrain collaboration. Authors recommend researcher reflexivity and awareness to mitigate these unequal dynamics.

###### Time

Time is mentioned as a constraint where it created tension around competing priorities [53], and where flexibility around time frames needed to be built into projects to manage unexpected events [39,50,51].

###### Tension around competing priorities

– Artz (2017) describes the Z-Card project where an NGO was required to commit time and capacity to engage substantively with revised versions of the card but had insufficient time to do this while continuing to do their core day-to-day work of clinical service provision.

###### Flexibility around time frames

– Unexpected issues meant that more time was needed for projects than initially anticipated. These included logistical issues related to working in an LMIC setting (poor road infrastructure) [39], challenges in participant recruitment [50], and delayed community buy-in [39,50,51].

###### Human Resources

Training for facilitators and mentors is mentioned by *n* = 11 projects [49–51,55,58,62–64,66]. Recruitment of mentors, facilitators and project staff is briefly mentioned by (*n* = 8) [39,50,51,54,58,62–66], for example where studies state that projects recruited local community members as mentors. Remuneration (for example stipends) is mentioned by (*n* = 3) projects [50,51,64,65]. Other aspects of human resources such as communication, performance management and staff turnover are not mentioned.

#### Power dynamics

Power dynamics were explicitly mentioned in (*n* = 11) projects. At macro level, unequal power between global funding structures and communities, undermined co-created project objectives [60]. At meso level, researchers and project partners should consider power dynamics as determinants of partnership functioning and reflect on their own positionality relative to other project participants [58,60]. They should also consider ways to equalise power, for example by giving the community control of resource allocation within the project [62]. Within communities, power dynamics underpinned attitudes to female mobility, GBV and reproductive health [50,51,63,64,66]. Researchers described a need to consider power dynamics within communities to avoid perpetuating inequality, for example, by reinforcing hegemonic norms where male promotores became community GBV experts in Peru [66]. At micro level men and husbands had greater power and decision-making control than women and girls [50,51,60]. Power could be shifted where participants claimed space by exercising collective power [50,51], as in the Parivartan Project, where girls achieved greater agency as participants in sport and public life. Projects also described shifting power – where this was held by researchers, men, community leaders, and elders – to women and marginalised groups through their action as collaborators and decision-makers [39,48,49,56,59]. Articles do not categorise types of power or conduct a formal power analysis.

## Discussion

This scoping review has systematically mapped published literature to understand what can be learned by examining gender and health programming undertaken within participatory partnerships in LMIC settings. While previous reviews have examined gender transformative or responsive health promotion for different groups [31,67–69], the intersection of participatory design and partnership processes in gender and health programming remains underexplored. A key finding in this scoping review is that a gendered perspective was brought to bear on a range of health issues, using varying forms of analysis and strategies to mitigate resistance. Overall, projects did not address structural or environmental drivers of inequality. Participatory approaches were shown to complement gender programming by providing iterative spaces for reflection, adaptation, and power-shifting; thereby fostering stakeholder agency and buy-in. However, these benefits rely on donor flexibility, as rigid funding and accountability frameworks can constrain participatory processes. Partnership dynamics were infrequently reported, representing a knowledge gap. Power dynamics emerged as a cross-cutting theme - influencing participation, implementation, and sustainability.

### Applying a gender lens in health programming

Projects employed gender programming across diverse health domains. These ranged from sexual and reproductive health and violence prevention, to nutrition, mental health, and COVID-19 risk mitigation – reinforcing that gender is an intersectional determinant of health. Yet, most actions labelled ‘gender-transformative’ were confined to individual or community-level norm shifting and capacity strengthening, with limited policy engagement or involvement of government actors. This aligns with broader literature emphasising that genuine transformation must extend beyond awareness-raising to tackle structural power relations [31,70,71]. To achieve sustainable change, health programmes should embed measurement frameworks that track gender transformation across multiple levels and integrate explicit engagement with policy and health system actors.

### Participatory design for gender programming

Most initiatives were co-designed rather than co-created, typically involving external actors who defined problems and communities who co-developed solutions. Although co-creation may yield deeper contextual relevance, it is harder to plan and fund within conventional donor structures that require predefined objectives and timelines. Donor inflexibility, therefore, remains a barrier to community-driven innovation [72].

Participatory design created opportunities for critical reflection and collective action, echoing Freire’s concept of praxis – reflection and action upon the world to transform it [73,74]. Such processes helped participants question power and gender norms while iteratively adapting interventions to local realities. Sustained stakeholder dialogue was particularly valuable in mitigating resistance – a known challenge in gender equality initiatives [11,75]. Building community trust, identifying potential backlash, and co-developing mitigation strategies with local actors can enhance programme legitimacy and long-term impact. To enable this, donors should establish funding mechanisms that accommodate flexible, iterative participatory approaches [76].

### Partnerships elements in collaborative gender programming

Despite the centrality of partnerships to participatory design supporting gender and health programming, most studies provided only brief descriptions of partner roles, decision-making, or conflict management. The absence of detailed reporting on partnership functioning – including trust, communication, and accountability – limits understanding of how collaboration enables or constrains gender transformation. Human resource aspects related to training were mentioned fairly often, highlighting their importance in sustaining equitable practice [77]. However, relational factors such as organisational hierarchies or conflicting institutional priorities were rarely explored, despite evidence that these shape power and learning in collaboration [78].

Future participatory gender programming should incorporate monitoring and evaluation frameworks that track relational dynamics, including conflict resolution, trust-building, and decision-making equity. These insights are critical for developing partnership models that strengthen gender-transformative capacity within health systems.

### Power as a cross-cutting element

Power dynamics were evident across macro, meso, and micro levels. At the macro level, donor–community relationships often reflected structural inequities, reinforcing hierarchies noted elsewhere [79]. At the meso level, organisational power relations influenced whose priorities shaped interventions. At the micro level, male dominance in decision-making sometimes persisted, and in some humanitarian settings, gender-norm interventions inadvertently reinforced patriarchal control. Such findings underscore the need for intentional power analysis within project design and evaluation – to ensure that participation is not tokenistic but truly redistributive of voice and influence. Gender-responsive frameworks offer practical tools for tracking these dynamics and could be integrated into project monitoring and evaluation systems [80,81]. By addressing power explicitly, participatory partnerships can move beyond programmatic engagement towards health system transformation – challenging hierarchical norms and strengthening responsiveness to gendered needs.

Although most projects operated at programmatic levels, their participatory mechanisms – reflection, local adaptation, and community ownership – represent key attributes of a learning health system. Embedding participatory and gender-transformative principles within policy and governance processes could enhance system responsiveness, accountability, and sustainability. Participatory partnerships contribute to health system strengthening by aligning equity goals with collaborative structures and adaptive learning processes.

#### Strengths and limitations

This review has some limitations. It includes original journal articles published only in English, not grey or unpublished literature. This was done to get a sense of what research in published work is available to inform projects. Many articles provided limited detail on internal partnership dynamics, human resource strategies, or the specific capacities required to implement gendered health approaches, participatory processes or partnerships involved. This constrained our ability, in some instances, to explore elements which shaped partnerships and how these were linked to participation and gender programming. Analysis was based on journal articles, which may be subject to positive bias or selective reporting, and which tend not to mention negative aspects of projects. There may be multiple articles describing projects, and this review may not have included all literature, but it is intended to give a broad overview. The search was conducted at the end of 2023 so subsequent studies may have been published which could be the subject of future research. This review is strengthened by clear conceptual framing and methodological rigour, providing fresh insight into the intersections of gender programming, participatory approaches, and partnerships. It identifies actionable gaps for future research.

## Conclusions

This scoping review found that participatory partnerships can strengthen gender programming in health by fostering reflection, adaptation, and opportunities for power shifting while enhancing community ownership. However, most initiatives operated at individual or community levels, with limited structural engagement, weak policy influence, and little government participation. Partnership processes – including collaboration, conflict, and power-sharing – were seldom documented, revealing a critical evidence gap.

Power emerged as a determinant across macro, meso, and micro levels, shaping how projects were funded, governed, and experienced. To achieve genuine gender transformation, future health programmes must integrate explicit power analysis, adaptive funding models, and robust partnership frameworks. Health systems and donor mechanisms should embed participatory and gender-transformative principles to support flexible, iterative, and equitable design processes. Doing so will enable participatory approaches to contribute not only to more inclusive programming also but to structurally responsive and gender-equitable health systems capable of sustaining change at scale.

## Supplementary Material

PRISMA SCR_Reporting Guidelines.docx

Supplementary_Material_Combined_clean.docx

## Data Availability

Datasets generated and analysed for this scoping review are available in the University of the Western Cape’s UWC Data repository: https://uwcscholar.uwc.ac.za/items/738301f3-82d8-4bb7-85c0-b6375bde8fe9.
