# Supplementary material for: Participatory design partnerships for gender and health in low- and middle-income countries: a scoping review
Source: Glob Health Action. 2026 Apr 23;19(1):2627106. doi: 10.1080/16549716.2026.2627106 (PMC13107990; doi:10.1080/16549716.2026.2627106)
Supplement: Supplementary_Material_Combined_clean.docx [file ZGHA_A_2627106_SM8067.docx]

**Supplementary File 1: Search strategy**

- Several articles were searched for terms related to co-design and gender, to develop a list of concepts related to the research question.
- “Gender” as a term on its own returned the same number of results as using it in combination with other terms. “Masculinity” was added after reading literature
- “NOT” was used to exclude articles unlikely to be related to gender mainstreaming such as dysphoria, reassign, "gender variance" or transgender

| **CONCEPTS:** | **Gender-related terms** | **Co-design related terms** |
| --- | --- | --- |
| **Sources** |  |  |
| (Pearce et al., 2020)  (Vargas et al., 2022)  (Masterson et al., 2022)  (Bazzano et al., 2017) |  | co-creation OR  co-assessment OR co-commissioning OR co-conception OR co-construction OR co-delivery OR co-design OR co-development OR co-dissemination OR co-evaluation OR co-ideation OR co-implementation OR co-initiation OR co-innovation OR co-learning OR co-management OR co-planning OR co-possibility OR co-production OR co-testing OR  knowledge creation OR knowledge co-production OR participatory |
| (Stewart et al., 2021)  (Rietveld et al., 2022) | “Gender”  “Gender* Stereotype*”  “Gender* Norm*”  “Gender Role”  “Gender Equal”  “Gender unequal”  “Gender Transformative”  “Gender sensitiv*”  “Gender responsive”  “Gender equity”  Gender inequity”  “gender-based”  Masculinity |  |

**SEARCH TERMS**

| String 1 (with truncation - *) | “co-crea*” OR “co-construc*” OR “co-deliver” OR “co-design” OR “co-develop*” OR “co-idea*” OR “co-implementa*” OR “co-initia*” OR “co-innova*” OR “co-learn*” OR “co-plan*” OR “co-produc*” OR “knowledge crea*” OR “knowledge co-produc*” OR participatory |
| --- | --- |
| String 2 (with truncation - *) | gender OR Masculinit* OR intersectional* OR feminis* OR patriarch* OR “Gender* stereotype*” OR “Gender* Norm*” OR “Gender Role” OR “Gender Equal” OR “Gender unequal” OR “Gender Transform*” OR “Gender sensitiv*” OR “Gender responsiv” OR  “Gender equit*” OR “Gender inequit” OR “gender-based” OR “gender mainstream*” OR “gender integration” OR “gender behavio*” OR “gender attitud*” |
| NOT | dysphoria OR reassign* OR "gender variance" OR transgender |

| **Database** | **Results** | **Filters** |
| --- | --- | --- |
| Search date: 21 November 2023 | | |
| **Web of Science** | 1857 | 2003-2023, English, Journal articles, filtered to exclude categories like computer science, geography, chemistry, engineering |
| **PUBMED** | 448 | 2003-2023, English, Journal articles |
| **Scopus** | 3709 | Limited to: Social, science, medicine, arts & humanities, environmental science, psychology, nursing, health professions, biochemistry, genetics and molecular medicine, multidisciplinary, immunology & microbiology, neuroscience, dentistry |
| Search date: 5 December 2023 | | |
| **CINHAL** | 432 | 2003-2023, English, Journal articles |

**Supplementary File 2: Abstract Screening Tool**

**Process -**

1. Sort the articles by Author in Covidence, to ensure we are screening in roughly the same order
2. Tag HIC/LMIC
3. Follow the guidelines below

**Tool –**

EXCLUDE AT FIRST “NO”

1. Are the Title and abstract in English?

*If no – EXCLUDE*

1. Is it published from 2003 onwards?

*If no – EXCLUDE*

1. Is the article related to health (description below)

*If no – EXCLUDE*

*If unclear – MAYBE & tag:* **Uncertain if health promotion?**

1. Is the article about efforts to improve gender norms, address gender-related attitudes or behaviour, the use of a gender transformative approach, gender mainstreaming, gender integration etc

*If no – EXCLUDE*

1. Does the article describe a collaborative/participatory design partnership
2. *If no – EXCLUDE*
3. If abstract cannot be accessed but the title does not exclude the article

*Vote MAYBE, and add a note*

**TAG -**

- *Uncertain if health promotion?*
- *HIC/LMIC*

**Definitions of health/health programme**

- A programme that successfully addressed a health issue (as defined by the programme) and can include - but is not limited to - changes in service utilisation, access to health services and resources, service provision, outreach, health knowledge, issues related to health workforce, health financing, etc.
- A programme that successfully responded to the needs and/or situations of particular gender groups (women, men, or non-binary people) **and** addressed power inequalities or harmful gender norms (e.g., transforming gender norms to abandon and end female genital mutilation, transforming power relations within the health workforce).
- contribute to improved health outcomes, which relate to and are not limited to community mobilisation, water and sanitation, nutrition, regulation of food, tobacco and alcohol, as well as health service delivery, health information systems, access to essential medicines, health workforce, health financing, leadership and governance.
- Sexual and reproductive health, mental health

<https://www.who.int/westernpacific/about/how-we-work/programmes/health-promotion#:~:text=Health%20promotion%20is%20the%20process,of%20social%20and%20environmental%20interventions>.

**Supplementary File 3: Extraction table and samples**

**Study Description:**

**Project Elements:**

**Supplementary file 4: Initial project plans and flexible adaptation**

| Projects identifier: First author of papers, year | Initial Plan | Flexibility and iterative adaptation |
| --- | --- | --- |
| Ahlberg, 2015 | The project began as an extension of an earlier HIV/AIDS prevention study. The initial plan was to respond to Naserian Primary School’s request for support with tree planting, to use the “a child, a tree” concept as an entry point for collaboration, to involve pupils, teachers, and parents in planting and caring for trees, and to integrate this activity into broader health promotion and community dialogue.  Tree planting was intended as a practical environmental activity that would create a platform for deeper engagement around health, HIV prevention, and social issues. | Although the project had no formal phases, it evolved in response to contextual realities:  **1. Unplanned shifts due to water scarcity**   - Severe water shortages and the failure of existing water sources meant tree survival was at risk. This prompted consultation with an engineer and the exploration of three new options (additional tanks, borehole, or water pan). - The water pan became a new focal point, re-shaping the project’s direction toward infrastructure and community water access, which had not been part of the original plan.   **2.Changes in participation patterns**   - Initially, parents participated jointly in discussions; however, the next day they insisted on gender-segregated groups, requiring the researchers to adapt facilitation plans on the spot. This unanticipated shift revealed underlying gender power dynamics and required a flexible response.   **3.Adapting to donor and NGO dynamics**   - Donor requirements forced the researchers into partnership with an NGO whose priorities conflicted with the collaborative approach. This created tension and required renegotiation, slowing progress and altering expected timelines. When donor timelines ended, the community continued elements of the tree-planting initiative independently, showing an unexpected spin-off and sustained ownership beyond the research team’s control.   **4.Iterative learning despite scattered field presence**   - The dispersed research team could not visit together, but staggered visits and remote communication maintained continuity. These ongoing check-ins enabled incremental learning, even though iteration was not formally structured.   **5.Evolving focus of project activities**   - Tree planting expanded from a symbolic educational exercise to a community-driven platform for interschool engagement, discussions about water infrastructure, community dialogue on HIV, health, and social issues. |
| Artz, 2017 | The Z Card project was a feminist, collaborative research initiative between a university research unit and four NGOs providing services to survivors of sexual violence.  The original plan was to   - to collect quantitative and qualitative data on service provision, police response, and survivors’ experiences; - to pilot the Z-Card, a printed guide designed to give survivors essential information to navigate health and criminal justice processes; - to distribute the Z-Card through partner NGOs, who would:   - hand out traceable cards during initial counselling sessions;   - track their movement during follow-up visits;   - enrol 350 survivors for telephone follow-up interviews about the card’s usefulness.   The intention was that the researchers would produce and supply the Z-Cards, gather evidence of impact, and advocate for broader distribution. | Although no formal iterative phases were described, the project had to be adaptively modified in several ways due to contextual constraints and ethical concerns.  **1. Recruitment and data collection strategy had to be abandoned**  The initial plan for 350 telephone follow-up interviews became unworkable due to:   - non-functioning or unreachable phone numbers, - survivors’ limited availability due to crisis response timelines, - NGOs’ heavy caseloads and limited time with clients, - concerns about re-traumatisation and ethical risks of intrusive questioning.   The team considered collecting data at the point of Z-Card distribution in medical facilities but determined that this would be ethically inappropriate because survivors are often in acute distress. Ultimately, the Z-Card pilot phase was withdrawn because evidence gathering was not feasible without compromising survivor safety.  **2. Flexibility was required to respond to NGO capacity constraints**  Partner NGOs struggled to distribute Z-Cards consistently, monitor their usage, recruit participants, collect required data.  The researchers accepted that frontline service priorities superseded research demands. The project shifted towards reflective learning about feminist collaboration rather than producing the planned dataset.  **3. Ethical considerations reshaped the project’s direction**  Concerns emerged around:   - imposing extra burden on traumatised survivors, - requiring frontline NGO staff to perform additional documentation, - risking further stress during a sensitive medical/legal process.   The researchers prioritised the ethical principle of “do no harm” and allowed survivors’ wellbeing and NGO constraints to guide the decision to scale back research activities |
| Bankar, 2018  Collumbien, 2019 | The Parivartan project aimed to determine whether a girl-centred sport programme could challenge restrictive gender norms and empower adolescent girls and promote girls’ mobility, confidence, leadership, and agency.  Initial design components:   - Engage community members (girls, mothers, fathers) through participatory research (problem trees, safety mapping). - Select an appropriate sport based on community input (kabaddi). - Train ten young women mentors to guide teams of athletes. - Deliver a 15-month curriculum combining:   - weekly kabaddi practice sessions,   - reflection and life-skills sessions,   - gender curriculum sessions focusing on equality, mobility, safety, and bodily autonomy. - Monitor progress through prospective qualitative case studies with mentors and athletes at three time points.   **Pre-planned structures**   - Regular attendance tracking. - Monthly mentor meetings to identify and resolve challenges. - A community advisory board for feedback and community endorsement. | **1. Curriculum adapted week-by-week based on real experiences**   - Topics introduced one week were tested through role play and games the following week. - Mentors modified how they facilitated sessions depending on girls’ reactions and comfort levels. - Sessions were adjusted when sensitive issues arose, integrating new content based on emerging needs.   **2. Ongoing stakeholder engagement guided programme changes**   - Monthly mentor meetings captured challenges (e.g., mobility restrictions, parental objections). - Regular discussions with parents and community members revealed new barriers and enabled responsive problem-solving. - Home-based engagement with fathers emerged organically as a safer space for men to observe their daughters’ participation.   **3. Safety concerns and gender norms led to adaptations in implementation**   - Girls’ mobility restrictions required careful negotiation of “respectable identities”. - Mentors collectively took risks as a group to normalise girls’ presence in public spaces. - Parents’ concerns about safety led to changes in supervision and timing of sessions. - Sensitive topics were introduced gradually once trust was established.   **4. Family engagement strategies evolved**   - Mothers emerged as key partners in negotiating increased freedom for girls. - Parent reflection groups expanded in focus after emotional responses to daughters' aspirations. - Engagement with fathers became more indirect to avoid backlash, occurring via mothers or daughters rather than group sessions.   **5. Mentor roles evolved organically**   - Mentors became cultural intermediaries between girls and parents. - They adapted activities in real time, based on safety issues, attendance patterns, and girls’ emotional needs. - Mentors’ own identities and experiences became a critical mechanism for norm-shifting.   **6. Community endorsement strategies adapted over time**   - Public performances and kabaddi matches were added strategically to build legitimacy. - Advisory board consultations informed changes to content and implementation pace. - Community visibility increased gradually as risk to girls decreased. |
| Beeman, 2023 | This project used human-centered design (HCD) to co-design dedicated menstrual health spaces—“Cocoon Minis”—for people who menstruate in the Bidi Bidi refugee settlement. The initial plan was intentionally structured and phased:  **Planned HCD Phases**   1. **Background research**    - Understand cultural and infrastructural challenges    - Review existing evidence, especially regarding menstrual product disposal    - Conduct stakeholder interviews 2. **Design research**    - Conduct interviews, FGDs, card-sorting, journey mapping    - Explore cultural norms, daily challenges, desired MH solutions 3. **Rough prototyping**    - Create four physical and “software” prototypes    - Test acceptability, feasibility, desirability with participants 4. **Live prototyping**    - Advance best prototypes    - Test high-resolution models over several weeks    - Analyse material supply chains and usage patterns 5. **Pilot phase**    - Construct 20 Minis in two settlement zones    - Conduct structured interviews with people who menstruate, community members, and Mini supervisors    - Assess damage, usage, acceptability, and feasibility for scale-up | **1.Insights from early phases reshaped design priorities**  Participants emphasised:   - lack of menstrual education, misinformation among men and boys, stigma around product disposal, lack of private, safe physical spaces.   **2.Prototype features were adapted through feedback cycles**  During rough and live prototyping:   - Physical structures were modified based on participants’ reactions to scale, privacy, ventilation, lighting, and safety. - Software components (education, sensitization activities) were revised in response to participants’ ideas about acceptability and cultural norms. - Participants tested physical models and role-played how the spaces would be used, leading to refinements in layout, privacy mechanisms, and disposal features.   **3.Sensitization efforts were introduced and expanded during implementation**  Sensitivity to community perceptions led to continued and expanded efforts:   - Kuja Kuja staff hosted ongoing sensitization meetings to build trust (not part of the original fixed plan). - These meetings helped maintain buy-in and reduce stigma risks around the Minis. - They continued throughout the project, not only in early phases.   **4.Engagement with men evolved unexpectedly**  Initially, menstrual spaces were designed only for people who menstruate. However:   - It became clear that stigma persisted because men associated menstruation with shame. - To address this, men were drawn into construction roles (digging trenches, backfilling, foundation work, wall-building). - This engagement helped men learn about women’s health issues indirectly, supporting buy-in.   **5.A storytelling and education event was piloted but discontinued**  The team tested a monthly community storytelling event to promote open dialogue about menstruation.  However:   - COVID-19 restrictions prevented continuation of these gatherings. - The team flexibly halted in-person events and focused instead on household-level sensitization and smaller group interactions facilitated by community workers.   **6.Pilot findings led to adaptations for future scale-up**  These revealed that some people who menstruate were reluctant to use the Minis if men were nearby, stigma could still cause non-use, and different settlement zones required slightly adapted designs.  These findings prompted modifications in recommendations for future iterations, such as:   - continuing gender-segregated education, adjusting Mini placement for privacy,   enhancing disposal system features, increasing community awareness activities. |
| Chowdhary, 2018 | The AMAL Initiative (“Adolescent Mothers against All Odds”) was designed as a multi-component, evidence-informed programme to elevate the voices of married and pregnant adolescents and first-time mothers in northwest Syria and make reproductive healthcare more adolescent-responsive.  It aimed to meet the immediate SRH needs of pregnant adolescents and first-time mothers in a conflict-affected setting, address gender, power, and social norms shaping early marriage and reproductive decision-making and create structures for adolescent participation and leadership in programme design, delivery, and evaluation.  This was done through -   - **Young Mothers’ Clubs (YMCs)**:   - Eight-session cycles with adolescents, covering contraception, birth planning, safe pregnancy, postpartum and newborn care, violence prevention, communication, and critical thinking. - **Community dialogue groups**:   - Mothers, mothers-in-law, husbands, religious and community leaders).   - Seven-session cycles on puberty, early marriage, power relations, GBV, family planning, and household decision-making. - **Health provider groups**:   - Discussed rights-based family planning, adolescent-friendly services, communication, and counselling.   **Participatory structures included -**   - **Adolescent Advisory Committees (AACs)**:   - YMC graduates who showed interest in leadership receive further training.   - AAC members identify hard-to-reach adolescents, share recommendations with stakeholders, and provide ongoing programme feedback in monthly meetings. - **Community Advisory Groups**:   - Select community members, providers, and AAC members acting as co-evaluators of the programme. | **1.Iterative participatory development of the curriculum**   - The initiative adapted global evidence-based approaches (CARE, UNICEF, Save the Children) for the Syrian humanitarian context through an “iterative participatory development process.” - Feedback from adolescents, families, providers, and local partners informed how global tools were tailored (e.g. topics, framing, examples, and facilitation strategies appropriate to local norms and conflict conditions).   **2.Session-level feedback loops and continuous quality improvement**   - At the end of each session in a cycle, facilitators conducted group reflection “session evaluations” to gather what went well, what did not, and suggestions for improvement. These reflections were used to adjust the way sensitive topics were introduced, pacing and depth of discussions, facilitation styles and examples used.   This was used for ongoing quality improvement.  **3.Adolescent Advisory Committees as an iterative design & evaluation mechanism**   - 33 YMC graduates joined AACs, receiving additional leadership training. Members identified marginalised and hard-to-reach adolescents to bring into programming, improving programme “reach”. |
| Doan, 2022 | The project aimed to improve PrEP uptake among transgender women in Vietnam by integrating gender-affirming care into PrEP and primary healthcare (PHC) services, using a Plan-Do-Study-Act (PDSA) continuous quality improvement (CQI) approach. | The project applied a continuous quality improvement (PDSA) approach, but very few iterative changes were explicitly documented in the article. |
| Forbes-Genade, 2019 | The GIRRL Programme used Participatory Action Research approach to help reduce the vulnerability of adolescent girls in a resource-poor setting by cocreating a programme of strategic activities for a more inclusive disaster risk reduction environment.  This project supported adolescent girls to identify and act on issues affecting their safety, health, and rights within their communities. Through facilitated workshops and collective inquiry, girls explored the root causes of gender-based violence, poverty, and marginalization, and designed community actions to challenge these issues. The intervention placed girls in leadership roles, promoted critical consciousness, and fostered solidarity, resilience, and advocacy skills. | **1.Stakeholder engagement evolved over time**   - The programme initially relied on community “gatekeepers” to identify issues. - As trust developed, girls increasingly took the lead in defining priorities.   **2.Curriculum content changed based on girls’ inputs**   - Although initial topics were suggested by stakeholders, girls later reshaped sessions to focus on peer pressure, pregnancy, disease stigma, lack of belonging   **3.The Action Plan emerged organically from reflection activities**   - Not planned at the outset—developed through brainstorming during sessions. It involved 1) a community awareness event (dance, drama, poetry conveying messages about safety and vulnerability) and 2) community-based activities led by girls (e.g., fire safety education, peer support sessions at youth centres)   **4.Reflection phases directly informed programme changes**   - The reflection stages provided space to critique the ethics and design of the programme; helped researchers plan improvements for later phases. |
| James, 2021 | The project aimed to co-design and test two community-based IPV interventions in humanitarian settings in Malaysia and Lebanon through planned stages, to co-develop a poster campaign. The programme engaged both women and men in gender-segregated and mixed sessions, using facilitated discussions to unpack harmful norms, explore power dynamics, and build empathy and communication skills. | The project involved collaborative development of workshop materials and facilitator tools, but does not describe iterative changes during implementation. |
| Kerr, Patel | The Soils, Food and Healthy Communities (SFHC) programme in Malawi began in 2000 as a farmer participatory research initiative intended to improve food security, improve child nutrition, and address gender inequality. Gender issues were recognised early but were a secondary aim at the outset of the project | **1. Home visits were initially planned as the core intervention**.  However, monitoring and feedback showed:   - Men did not participate. Women experienced increased workload. - Benefits were limited. Improved crop availability did not automatically improve nutrition.   **2.Co-development of a new collective format : Recipe Days**  Through discussions among project staff, researchers, and farmers Recipe Days were created collaboratively. This emerged from community reflection and allowed both sexes to cook, discuss, and renegotiate gender roles in a public space.  **First Recipe Day = pilot test**   - Only 20 participants; few men who were uncomfortable performing cooking tasks. Participants discussed modifications.   **3. Scaling up and modifying format based on participant feedback**  After the first Recipe Day, a technical workshop led to:   - A decision to hold larger community meetings. - Inclusion of both men and women, with public discussion of gender norms. - Joint cooking of multiple dishes. - Discussions on workload, decision-making, and food allocation.   **4. Strengthening gender-transformative elements over time**  As Recipe Days evolved:   - Men began to openly reflect on their gendered behaviours. - Women gained public platforms to discuss unequal workloads. - Men and women jointly performed tasks that challenged gender norms. |
| Kutwayo, 2018 | The Girls Achieve Power (GAP) Year intervention aimed plan, implement and evaluate a curriculum with stakeholders including adolescents, parents, teachers, health workers, and government officials to empower adolescent girls and shift gender norms by improving their educational, health, social, and economic potential | **1.Adjustments based on stakeholder mapping and engagement feedback**  Initial engagement only partially reached school staff, so the team conducted:   - follow-up visits to secure school buy-in, additional meetings with principals, - school-level clarifications and trust-building activities.   School staff raised concerns about absenteeism linked to lack of sanitary pads, gang involvement, pregnancy rates.  These new concerns led the project team to:   - revisit baseline data, adjust planned activities, consider sanitary pad provision, refine the curriculum to address newly surfaced issues.   **2.Continuous curriculum modification driven by adolescents and coaches**  Iteration was visible in curriculum adjustments:   - Coaches assessed feasibility of activities in their contexts. - Curriculum developers adapted materials (images, terminology, topics). - Adolescents contributed topics they felt were missing (e.g., mental health).   This resulted in:   - redesigned session content, revision of key messages, age-appropriate and context-specific language, inclusion of themes that were not part of the original blueprint.   **3. Iterative contextual tailoring**  Coaches acted as cultural/contextual intermediaries:   - They assessed feasibility of activities in each school context. - The curriculum developer made amendments accordingly. - Parents provided feedback on acceptability of policy content. - Principals, as gatekeepers, influenced pacing and implementation logistics.   This meant the intervention could not be implemented identically across schools; instead, it evolved based on local realities. |
| Malta, 2023 | The Malta study aimed to develop, refine, and test a mobile health intervention (the Rainbow Resistance—Dandarah app) to address violence and discrimination against LGBTQ+ / SGM persons in Brazil. The planned steps were:  1. Conduct formative research  2.Analyse qualitative data  3. Develop the first version of the app  4. Conduct a pilot test  5. Finalise the app for public release | Although not framed using formal cycles, the study provides clear evidence of iterative and flexible co-design informed throughout by the SGM community.  Three major forms of iteration are evident:  **1.Continuous adaptation guided by community insights (FGDs, IDIs, CAB)**   - The qualitative data directly shaped app features (e.g., panic button, violence mapping, support pathways).   - CAB members representing diverse SGM subgroups met monthly to adapt recruitment strategies, refine study materials, advise on culturally sensitive language, interpret findings, shape iterative adjustments.   **2.Pilot-testing**  After ten SGM participants tested the prototype:   - Users gave feedback about comprehension, usability, feature relevance, and overall experience. The research team reviewed all suggestions. App features were revised and improved before the public launch. - A dedicated “features-tailoring round” occurred based on pilot results.   **3. Flexibility in response to differences within the SGM community**  The study explicitly describes adjustments made for different subpopulations. For example:   - Trans participants had different needs and concerns than LGB participants. - The team adapted testing sessions and app functions accordingly. Participants noted that this differentiated approach signalled respect and relevance (“we did different things because the needs…are completely different”). |
| Mauka, 2021 | This project aimed to develop, test, and refine a mobile health application to support HIV PrEP uptake and adherence among key populations, particularly men who have sex with men (MSM) and female sex workers (FSW), in urban East Africa.  The project followed the Information Systems Research (ISR) framework with three structured cycles:  1.Relevance Cycle (Problem identification and justification)  2.Design Cycle (Developing the app prototype)  3. Rigour Cycle (Piloting and refinement) | **1.Continuous consultation and refinement across all ISR cycles**  User contributions influenced each stage of the app’s evolution:   - In the relevance cycle, their perspectives shaped fundamental elements of the app’s perceived value. - In the design cycle, their preferences directly informed app architecture, data-saving features, and offline functionality. - In the rigour cycle, their feedback during FGDs and pilot testing led to concrete modifications.   **2.Prototype testing occurred in multiple stages and included user-driven modification**  Prototype refinement unfolded in explicit steps:   1. **Stage I testing by investigators**    - Preliminary review of essential functions.    - Integration of corrections for usability and relevance. 2. **Stage II testing by MSM and FSW participants**    - Review of different app logos and interface designs.    - Suggestions on terminology, safety, anonymity, and feature visibility.    - User feedback was systematically forwarded to developers for immediate modification.   **3. App features were directly modified in response to concerns about stigma, discrimination, and safety**  Participants emphasised:   - the need for anonymity, protection against legal and social risk, sensitivity in how target groups were labelled.   In response, investigators:   - replaced explicit identity labels (e.g., “MSM,” “FSW”) with **indistinct terms** to reduce risk, ensured offline accessibility for those with limited data resources, - emphasised data security measures.   **4. Iteration was also influenced by the broader structural context (stigma, criminalisation, technology constraints)**  The development team:   - Adjusted app design to accommodate intermittent internet access, - Considered compatibility limits with older mobile devices, - Managed recruitment challenges in criminalised populations using peer-led approaches.   **5. Post-pilot insights fed into further planned refinements**  Participants highlighted:   - desire for enhanced chat features with health workers, improved medication reminders, more intuitive navigation. |
| Moran, 2022 | This project used Participatory Action Research (PAR) to co-design a culturally appropriate COVID-19 risk communication and community engagement strategy with a rural, highly disadvantaged community in Northwest Pakistan. The project was structured around four predefined components:   1. PAR meetings with male and female community groups to identify challenges and develop strategies to reduce COVID-19 transmission. 2. Resource mobilisation based on needs identified by PAR members (e.g., access to water, sanitation). 3. Delivery of health messages by Community Health Champions (CHCs), using the WHO Family Toolbox to support families to map risky daily activities and modify behaviours. 4. Focus group discussions with wider community members to evaluate the intervention and its perceived impact. | Although the study had predefined steps, the PAR process led to adaptation in both the intervention content and implementation approach.  **1.PAR groups actively shaped the agenda**  Although each meeting had a planned structure, facilitators deliberately kept agendas flexible so PAR members could lead discussions. This shift enabled participants to bring forward issues not initially anticipated by researchers, such as:   - water scarcity, - lack of electricity to power pumps, - barriers to hygiene and sanitation.   As a result, the project expanded beyond communication strategies to include infrastructure-related solutions.  **2.Solutions evolved based on community-led decision making**  The PAR process began with behaviour-change messaging, but participants quickly identified structural constraints (limited water, unreliable electricity) that prevented adoption of preventive behaviours.  Through iterative discussions, the groups decided to:   - resource-mobilise for water pumps, pipes, and bores; propose installation of solar panels to power wells; integrate technical solutions with health communication.   **3. Trust-building processes shaped participation and inclusion**  The project adapted to patriarchal norms by forming gender-segregated groups, but it also leveraged existing family structures to overcome cultural barriers. Over time women reported increased confidence, women felt their opinions were valued at home, participation enabled women to exercise a public voice normally absent from decision-making forums.  **4. Integration with Pashtun cultural institutions strengthened legitimacy**  Researchers discovered alignment between PAR practices and Pashtun traditions of deliberative dialogue. Over the course of the project community leaders supported recruitment, CHCs shared findings back to the wider community, PAR discussions were linked with existing community dialogue spaces.  **5. Gender analysis became integrated through participation rather than planned assessment**  While no formal gender analysis framework was pre-specified, gender inequalities emerged through:   - women’s restricted movement in patriarchal settings, low literacy and limited access to information, women’s exclusion from traditional decision-making.   The project responded by:   - creating safe, women-only spaces, incorporating women’s insights into community-wide decision-making. |
| Pepper, 2023 | This project was initiated when a clinic facility manager approached a local NGO (Ubunye Foundation) due to rising numbers of postpartum women defaulting on antiretroviral therapy (ART). The initial plan was to:   - conduct a Photovoice-based Participatory Action Research (PAR) study with postpartum women living with HIV, - identify barriers to ART adherence through women’s photographs and narratives, - ensure co-researchers’ capacity by training women in Photovoice, camera use, ethical considerations, and weekly debrief processes, - analyse themes collaboratively between women co-researchers and Ubunye staff, and - use findings to inform advocacy abstracts and early programme planning. | **1. Women transitioned from “participants” to “researchers” to programme leaders**  While the initial plan involved women documenting their lived realities, the participatory process transformed their roles far beyond expectations:   - women acted as co-researchers, co-analysing data, validating interpretations, and agreeing final themes; - they co-designed abstracts and presented findings at national and international forums; - they led the design and implementation of interventions through Programme Working Groups (PWGs).   **2.Weekly debriefs created iterative cycles of reflection and adaptation**  The original design included weekly photo review sessions, but these sessions became a mechanism for ongoing adjustment:   - new themes surfaced that had not been anticipated by researchers; - co-researchers refined problem statements as their confidence and analytical insight grew.   **3. Photovoice findings directly shaped a multi-component intervention**  The project expanded beyond documentation and analysis to the co-creation of a comprehensive programme. The Programme Working Group (PWG), led partly by the women, identified four major action areas:   1. Individual: life-planning support with community health workers and Ubunye staff. 2. Community: open discussions about HIV stigma in schools and organisations. 3. Economic: life skills, mentoring, and business support to reduce dependence and vulnerability. 4. Peer support: confidential WhatsApp groups supported by mobile data and phones.   These interventions were not pre-specified; they emerged from iterative engagement and women’s priorities.  **4. Intersectional gender analysis emerged organically through the women’s contributions**  Although there was no formal “gender analysis” phase, women’s Photovoice data revealed intersecting systems of power shaping adherence. This perspective reshaped the programme to address structural and gendered barriers holistically rather than focusing narrowly on clinical adherence. |
| Stern, 2021 | The GAP Project engaged community activists in Peru and Rwanda in gender-based violence prevention. The Rwandan arm of the project did not feature participatory programme development and was therefore not included in this description. In Peru, phases included –  **1.Recruitment and training**   - Identified activists or “promotores” and supported them to design a set of prevention activities tailored to local GBV dynamics.   **2.Implementation of community prevention activities**   - Over six months, promotores implemented activities across multiple communities, and conducted household visits, community meetings, advocacy engagements,   **3. End-of-project participatory evaluation**  A final series of workshops was planned to evaluate prevention activities and promotores perceptions, to explore whether the approach could be used to generate contextually relevant prevention activities and mobilise communities around GBV prevention. | The PCID process was inherently iterative, and several unplanned adaptations emerged during implementation.  **1.Gendered participation constraints required ongoing adjustment**   - Promotores also had to navigate masculinity norms: men feared being viewed as “weak” for engaging in GBV prevention. This gendered dynamic was not fully anticipated and required promotores to develop sensitive communication strategies to mitigate backlash.   **2.Context-specific barriers led to iterative redesign of activities**   - activists had to work independently across geographically dispersed communities; trust-building took more time than expected; - household privacy norms made discussing violence publicly difficult.   Promotores developed adaptive strategies such as:   - approaching households with respect and humility rather than authority, - seeking legitimacy via local elders and government partners, - tailoring messaging to different audience types. |
| Taliep, 2023 | This project aimed to use Community Asset Mapping (CAM) to develop an interpersonal violence prevention programme in a low-income community in the Western Cape. The initial plan was not organised into formal phases but followed a series of structured steps:  **1. Initial catalyst for the intervention**   - Community representatives approached researchers to initiate a violence prevention intervention. - There was a recognised lack of research incorporating community assets into violence prevention.   **2. Community and service provider mapping workshops**  Two parallel workshop streams were planned:  Community workshops  Focused on:   - mapping tangible and intangible assets (e.g., interfaith networks, trust, respect), - identifying factors undermining peace and safety (substance abuse, unemployment, violence), - ranking organisations by contribution to safety, - exploring roles of spirituality and masculinities in violence and safety.   Service provider workshops  Focused on:   - mapping organisational footprints and services, - identifying overlapping mandates and service gaps, - mapping collaboration networks, - identifying spiritual assets and masculinities-related programming.   This information was used to as a basis for intervention planning.  **3. Prioritisation and intervention design**  In action planning workshops, participants selected one intervention focus using criteria such as:   - feasibility, alignment with CBPR principles, links to change theory, potential to mobilise community assets, focus on positive forms of masculinity.   The intended outcome was a mentoring programme that engaged adults as mentors and youth as mentees to model positive masculinities and promote safety and peace.  **4. Research with community members**  Community participants took on a role as co-researchers in the project. | **1. New priority areas emerged organically** through iterative ranking and dialogue.   - Masculinity emerged as a central theme unplanned at outset, driven by participant discussions. - Service providers identified gaps not visible to residents, influencing the intervention focus. - The original broad mapping approach narrowed to a targeted masculinity-focused mentoring intervention.   **2.Identification and mobilisation of partners was emergent**  The initial plan did not specify which partners would drive the mentoring programme. Iteration led to:   - Community participants singling out Hearts of Men (HOM) for addressing masculinity challenges. - HOM and the Cochoqua Cultural Council becoming key partners through the workshop process. - The Local Network of Care was not planned but evolved from recognising collaboration gaps.   **3. Power dynamics required constant reflexive adaptation**   - Tensions emerged between academics, community researchers, and residents. Researchers had to continually revisit their positionality, shifting from expert role to co-learner/co-producer.   **4. Training needs expanded as new roles emerged**  While the original plan anticipated community involvement in mapping and reflection, it became clear that community activist researchers needed additional training and researchers needed to adjust the analytical process to enable shared decision-making.  **5. Final programme was not predetermined but co-produced**  The final mentoring programme (later known as the Building Bridges Mentoring Programm5.emerged through community priority-setting, synthesis of service provider insights, application of socio-ecological and masculinity frameworks, iterative plenary discussions across multiple workshops. |
| Varjavandi, 2017 | AIM: The “#Blessers Must Fall programme implemented a youth-led participatory action research (YPAR) programme focused on teenage pregnancy, transactional sex (“blesser” relationships), and gender-based violence.  The programme followed a structured five-stage PAR cycle:   1. **Pre-reflection** – team building, identity exploration, negotiating differences of opinion and learning styles. 2. **Topic identification** – participants studied their social context and selected their focus problem. 3. **Data gathering & interpretation** – youth developed and administered short surveys with peers, family, and community members. 4. **Plan of action** – designing creative interventions such as drama, poetry, and photo-story posters. 5. **Implementation** – performing drama and sharing visual outputs at schools and Eco Club meetings. 6. **Evaluation** – ongoing reflective activities and end-of-week interview surveys. | Compared to other studies in the review, this project had a short time frame and limited iterative change. However, flexible adjustment included:  **1. Youth-driven topic selection**  Although facilitators introduced PAR and visual methods, the specific intervention focus was not predetermined. Participants independently identified teenage pregnancy and the “blesser” phenomenon.  **2. Creative methods evolved organically**  While the programme planned to include visual and participatory methods, the specific formats were shaped by youth choices.  For example:   - The first group chose to perform their drama and poetry at another school. - Facilitators then introduced the photo-story poster approach when participants wanted wider dissemination of their messages. - The second group then voluntarily replicated the drama/photo/story model for their theme.   **3. Implementation modalities shifted based on opportunities**   - Youth performed at another school (not originally planned). - Eco Club presentations were added as dissemination opportunities that emerged during the process. |

**Supplementary File 5:** **Evidence of joint ownership of project plans**

| **Author / Year / Country** | **Evidence of joint ownership of plans** | **How this strengthened contextual appropriateness** |
| --- | --- | --- |
| Ahlberg et al. 2015 – Uganda | Solutions were developed by the community, who identified issues which were relevant to them, and undertook sustained engagement and problem solving during the project despite men being less involved with HIV dialogues than was hoped. | Sustained community engagement gave rise to co-created solutions that addressed the local environment |
| Beeman et al. 2023 – Uganda | Community co-design sessions; local supervisors responsible for menstrual health structures. | Rooted in culturally relevant stigma concerns; tailored to safety and community maintenance needs. |
| Chowdhary et al. 2018 – Syria | Girls, families, and religious leaders co-created SRH and marriage solutions. | Reflected cultural and religious context; legitimacy through faith leaders. |
| Bankar et al., 2018, Collumbien et al. 2017 – India | Parents, mentors, NGOs, and girls jointly shaped sport-based programme. | Addressed parental safety/reputation concerns; adapted sports to local norms. |
| Forbes-Genade 2019 – South Africa | Developed by girls in the community, connected to community leaders. | Girls identified needs themselves, based on their local context |
| James et al. 2021 – Syria | Participants co-developed poster campaigns and awareness materials. | Tailored to local gender norms and IPV realities in conflict settings. |
| Kerr et al. 2016 – Malawi | Men and women jointly organised nutrition activities and recipe days. | Linked to local farming, food practices, and caregiving roles. |
| Patel & Kerr 2015 – Malawi | Recipe Days were planned and run by community members over a lengthy time period | The plan evolved from, and adapted in response to, community needs |
| Pepper 2023 – South Africa | Women were activists who implemented the program and took on advocacy roles | Women designed the project activities in response to challenges they identified in their own contexts |
| Stern et al. 2022 – Peru & Rwanda | Activists co-designed prevention strategies and symbolic acts. | Drew on local practices; legitimacy built through community dialogue. |
| Taliep et al. 2023 – South Africa | Community asset mapping engaged churches, CBOs, and leaders. | Grounded in existing community strengths; locally owned. |
| Varjavandi 2017 – South Africa | Youth used PAR, drama, and storytelling to define and act on priorities. | Interventions addressed lived experiences of GBV and transactional sex. |

Projects with less evidence of joint ownership of participatory design

| **Author / Year / Country** | **Participatory design with less evidence of joint ownership** |
| --- | --- |
| Artz et al. 2017 – South Africa | Intervention was largely NGO-driven. |
| Doan et al. 2022 – Vietnam | Focus on clinic/CBO staff training in gender-affirming HIV services. |
| Kutywayo 2018 – South Africa | Guidelines co-produced, but remained research-driven. |
| Malta 2023 – Brazil | Advocacy and training emphasised; interventions not co-created with communities. |
| Mauka 2021 – Tanzania | HIV mHealth app designed with input, but ownership stayed with implementers. |
| Moran 2022 – Pakistan | PAR shifted attitudes but didn’t document collective control of interventions. |

**Supplementary File 6: Gender profile of participants**

| **Author, year, country** | **Description of gendered engagement** |
| --- | --- |
| Ahlberg et al. 2015 – Uganda | Men were included as well as women, with the aim of engaging men in discussions on HIV prevention and sexual and reproductive health. |
| Artz et al. 2017 – South Africa | The gender of participants is not discussed, the paper describes involvement of survivors of sexual violence but does not distinguish between male and female survivors. It can be assumed that both were the intended beneficiaries but this is not stated. |
| Bankar et al. 2018, Collumbien et al. 2017 – India | Male family members (fathers, brothers) negotiated freedoms for female mentors; male community members as well as female were the intended audience for norms shifting. |
| Beeman et al. 2023 – Uganda | Male community members were included in co-design sessions and consultations to reduce stigma and support ownership of menstrual spaces. This article refers to people who menstruate, showing an inclusive approach to all genders. |
| Chowdhary et al. 2018 – Syria | Male family and community members (husbands, in-laws, leaders) participated in reflective dialogues on early marriage and family planning. |
| Doan et al. 2022 – Vietnam | Male engagement was indirect; the study focused on transgender women, with male partners included mainly as part of risk context. |
| Forbes-Genade 2019 – South Africa | Male community stakeholders indirectly involved through dialogues on resilience and disaster risk reduction. |
| James et al. 2021 – Syria | Men and women both engaged in 3-day workshops on IPV, gender norms, and help-seeking; men targeted to reduce acceptance of violence. |
| Kerr et al. 2016, Patel 2015 – Malawi | Male household heads were deliberately engaged in agroecological and nutrition dialogues to shift gendered power around food and caregiving. Men took part in in participatory nutrition education and cooking demonstrations, challenging gendered caregiving norms. |
| Kutywayo 2018 – South Africa | Males were not directly the focus but were considered in discussions around community norms and the role of male family members and leaders in enabling or constraining girls’ participation. |
| Malta 2023 – Brazil | Men (particularly cisgender men) were engaged indirectly as part of broader interventions targeting discrimination and violence. Male allies were encouraged to participate in advocacy against homophobia/transphobia. |
| Mauka 2021 – Tanzania, | Male partners were included as target users in some mHealth engagement strategies, particularly in encouraging testing and preventive behaviours. |
| Moran 2022 – Pakistan | Men and women were engaged as equal participants in participatory action research meetings, shifting from skepticism to being “agents of change.” |
| Pepper 2023 – South Africa | Male involvement was limited; the study focused mainly on post-partum women’s lived experience of HIV/AIDS stigma, violence, and support. |
| Stern 2022 – Peru | Male activists were intentionally included as community change agents, with women unable to take on the role of "promotores" in Peru as they did not have enough free time. Authors note this inadvertently positioned men as GBV "experts". |
| Taliep 2023 – South Africa | Male community members were engaged through a masculinity-focused lens, encouraging reflection on positive and non-violent masculinities. However, this project featured mixed-gender participation with women activists and researchers taking part and providing female perspectives. |
| Varjavandi 2017 – South Africa | This involved mixed genders, with both males and females engaged in participatory research and creative activities. They contributed perspectives on transactional sex and GBV. However, females were more well represented within the group, making up 17 of 20 participants |
